# Impact of short‐term Dutasteride treatment on prostate‐specific membrane antigen expression in a mouse xenograft model

**DOI:** 10.1002/cnr2.1418

**Published:** 2021-05-19

**Authors:** Benedikt Kranzbühler, Rosa Sousa, Lukas Prause, Irene A. Burger, Niels J. Rupp, Tullio Sulser, Souzan Salemi, Daniel Eberli

**Affiliations:** ^1^ Department of Urology, University Hospital Zürich University of Zürich, Laboratory for Urologic Oncology and Stem Cell Therapy Zürich Switzerland; ^2^ Department of Nuclear Medicine, University Hospital of Zürich University of Zürich Zürich Switzerland; ^3^ Department of Nuclear Medicine Kantonsspital Baden Baden Switzerland; ^4^ Department of Pathology and Molecular Pathology, University Hospital of Zürich University of Zürich Zürich Switzerland

**Keywords:** Dutasteride, prostate cancer, prostate‐specific membrane antigen

## Abstract

**Background:**

Dutasteride has been shown to increase expression of the prostate‐specific membrane antigen (PSMA) in prostate cancer cells in previous in vitro studies. This 5‐alpha‐reductase inhibitor is commonly used for the treatment of symptomatic benign prostatic enlargement. The modulation of PSMA expression might affect PSMA‐based prostate cancer imaging and therapy.

**Aim:**

The purpose of this work was to further analyze concentration‐dependent effects of Dutasteride on PSMA expression in a mouse xenograft model.

**Methods and results:**

Four groups of mice bearing LNCaP xenografts were treated for 14 days with daily intraperitoneal injections of either vehicle control or different concentrations of Dutasteride (0.1, 1, 10 mg/kg). Total expression of PSMA, androgen receptor (AR), and caspase‐3 protein was analyzed using immunoblotting (WES). In addition, PSMA, cleaved caspase‐3 and Ki‐67 expression was assessed and quantified by immunohistochemistry. Tumor size was measured by caliper on day 7 and 14, tumor weight was assessed following tissue harvesting.

The mean PSMA protein expression in mice increased significantly after treatment with 1 mg/kg (10‐fold) or 10 mg/kg (sixfold) of Dutasteride compared to vehicle control. The mean fluorescence intensity significantly increased by daily injections of 0.1 mg/kg Dutasteride (1.6‐fold) as well as 1 and 10 mg/kg Dutasteride (twofold). While the reduction in tumor volume following treatment with high concentrations of 10 mg/kg Dutasteride was nonsignificant, no changes in AR, caspase‐3, cleaved caspase‐3, and Ki‐67 expression were observed.

**Conclusion:**

Short‐term Dutasteride treatments with concentrations of 1 and 10 mg/kg significantly increase the total PSMA protein expression in a mouse LNCaP xenograft model. PSMA fluorescence intensity increases significantly even using lower daily concentrations of 0.1 mg/kg Dutasteride. Further investigations are needed to elucidate the impact of Dutasteride treatment on PSMA expression in patients.

## 1 INTRODUCTION

Prostate‐specific membrane antigen (PSMA)^1^ is a type II transmembrane glycoprotein which is highly expressed in 85%‐95% of all prostate cancer lesions.^2^, ^3^ Small molecules targeting PSMA are increasingly used for imaging and also therapy of advanced prostate cancer.^4^ However, the performance of PSMA targeting applications is directly correlated to the PSMA expression of the respective tumor tissue.^5^

Upregulation of cellular PSMA expression following androgen deprivation therapy (ADT) has initially been described in 1996 in primary tumor tissue samples.^6^ In vitro and in vivo studies confirmed an increased PSMA expression also following treatment with novel antiandrogens.^7^, ^8^, ^9^ In addition, retrospective and primary prospective studies suggested an altered PSMA uptake in patients following ADT or a direct androgen receptor (AR) blockade.^10^, ^11^, ^12^ Since the exact regulatory cascade involved is still unknown, in‐depth knowledge on function and regulation of PSMA may be used to increase the performance of PSMA‐based prostate cancer imaging and therapy.

In our previous research, we demonstrated that upregulation of PSMA expression can not only be achieved by the androgen receptor blocker Enzalutamide but also by high concentrations of Dutasteride in vitro.^13^ In addition, the effect of Dutasteride on PSMA expression was observed to be time‐dependent in LNCaP cells.^14^ Dutasteride, a 5‐alpha‐reductase inhibitor with a well‐tolerable risk profile is usually prescribed for the treatment of bladder outlet obstructions due to benign prostatic hyperplasia.^15^ Considering the low risk of severe side effects, Dutasteride is an interesting candidate to potentially induce PSMA expression prior to PSMA targeting applications.

In this study, we aimed to analyze concentration‐dependent effects of Dutasteride on PSMA expression in an LNCaP‐bearing mouse xenograft model. In addition, the impact on tumor growth and weight, AR expression, and apoptosis were analyzed.

## 2 MATERIALS AND METHODS

### 2.1 Cell culture and animal experiment

Prostate cancer cells (LNCaP, CRL‐1740) purchased from American Type Culture Collection (ATCC, Manassas, USA) were cultured in RPMI with phenol red (Life Technologies, ThermoFisher Scientific, Waltham, MA, USA) supplemented with 10% FBS and 1% penicillin/streptomycin (P/S). Cells were incubated at 37°C with 5% CO_2_.

Local animal ethical committee (Veterinäramt Zürich, Amendment to Licence No.244/2016) approved all experiments. A total of 16 nude mice (8 weeks old; male; Charles River Laboratories, Sulzfeld, Germany) were studied. Mice were subcutaneously inoculated on left and right backsides with 5 × 10^6^ cells (LNCaP) in a 1:1 mixture with high concentration Matrigel (500 μl, Corning Life Sciences, NY, USA) on each side. Tumor was generally palpable after 14 days. Afterward mice were divided into four groups: four animals were treated with daily intraperitoneal (i.p.) injection of vehicle (2% DMSO +30% PEG 300 + 2% Tween 80 + saline at 4 mg/ml), four with Dutasteride (0.1 mg/kg), four with Dutasteride (1 mg/kg), and four with Dutasteride (10 mg/kg) for 14 days (Selleckchem, Luzern, Switzerland). Tumor volume was measured by caliper (*V* = *L* x *W*2 /2) after 7 and 14 days. Following tissue harvesting, all samples were additionally assessed for tumor weight. One mouse in the 10 mg/kg group was excluded from final analysis due to preterm scarification caused by poor animal status.

### 2.2 Sample processing and immuno‐/histological assessment

All harvested tissue samples were divided into two pieces: one part was snap frozen for protein analysis, the other part was fixed in 10% buffered formalin (ThermoFisher Scientific, Waltham, MA) and subsequently processed and embedded in soft paraffin (Sargent‐Welch Scientific, Skokie, IL). Samples were cut at sections of 5 μm and further processed.

Hematoxylin and eosin (H&E) (Vector Laboratories, Burlingame, CA) stainings were performed according to the manufacturer's protocol. Paraffin‐embedded tumor samples were first deparaffinized by treatment with xylene and then rehydrated by passage through a graded series of ethanol. Indirect immunostainings were performed at 4°C overnight using the primary antibodies Anti‐PSMA/FOLH1 (clone 460 420, 1:100, R&D Systems, Zug, Switzerland), anti‐caspase 3, active (cleaved, AB3623, Merck, Switzerland), and anti‐Ki‐67 (AB9260, Merck, Switzerland). The slides were incubated with a secondary goat antirabbit FITC antibody (1:500, Vector Laboratories) or goat antirabbit Fluoprobes FP‐488 (Chemie Brunschwig, 1:500, Switzerland) or sheep antirabbit Cy3 (C2306, 1:500, Sigma Aldrich, Switzerland) at room temperature for 1 hour. After counter‐staining with DAPI (4′,6‐diamidino‐2‐phenylindole, Sigma‐Aldrich, 1:200), images were taken by confocal laser‐scanning microscopy (Leica SP8 inverse microscope, Mannheim, Germany). For negative controls, the primary antibody was omitted. For measurement of fluorescent intensity, cross sections from different animals and different experimental conditions were stained and images were taken by confocal microscope (n = 15‐20, 20× HPF). Fluorescent intensity was measured using Image J (1.4, NIH, USA).

### 2.3 Protein simple immunoblotting (WES)

Tumor samples were crushed in liquid nitrogen and resuspended in modified lysis buffer supplemented with a protease inhibitor cocktail (Sigma‐Aldrich, Buchs, Switzerland). Afterward, samples were centrifuged for 20 minutes at 13 000 rpm and the supernatant was collected for protein determination. Total protein was measured using a BCA protein assay kit (Thermo Scientific, Lausanne, Switzerland). Protein at a concentration of 1 mg/ml was used for the WES (automated western blotting). Sample preparation using the 12‐230 kDa cartridge kit was performed according to the manufacturer's protocol. Briefly, cell lysate containing 1.0 μg of protein was combined with a provided sample master mix consisting of 1× sample buffer, 1× fluorescent molecular weight marker and 40 mmol/L dithiothreitol (DDT). The samples were mixed and heated at 95°C for 5 minutes to denaturate the proteins and obtain a better protein separation. Then, individual glass microcapillaries were loaded with stacking and separation matrices followed by protein sample loading. During capillary electrophoresis, the proteins were separated by size and then immobilized to the capillary wall. The separated proteins were then combined with blocking solution and probed with primary antibody, horseradish peroxidase–conjugated secondary antibody and chemiluminescent substrate using the WES machine (ProteinSimple) and the 12‐230 kDa cartridge kit. Primary antibodies used were mouse anti‐PSMA/FOLH1 (R&D Systems, 4:100), rabbit anti‐AR (CellSignaling, 1:200), mouse anti‐caspase‐3 (Novus Biologicals Europe, 1:50). Mouse anti‐GAPDH (Novus Biologicals, 1:100) was served as internal control and analyzed using the Compass software (ProteinSimple). Virtual blot and electropherogram of each sample were checked and evaluated. A sharply defined chemiluminescent signal was quantified by the software, and the area of each sample was normalized to the GAPDH.

### 2.4 Statistical analysis

Data analysis was performed using GraphPad Prism (GraphPad Software, Inc., La Jolla, CA, version 7). Data were analyzed using a one‐way ANOVA with Bonferroni's multiple comparison post‐test or a Kruskal‐Wallis test with Dunn's multiple comparison posttest. Statistical significance was assumed if *P*‐values were < 0.05. Data presented is expressed as mean with corresponding SE of the mean.

## 3 RESULTS

### 3.1 Assessment of treatment response in a mouse xenograft model

**FIGURE 1:**
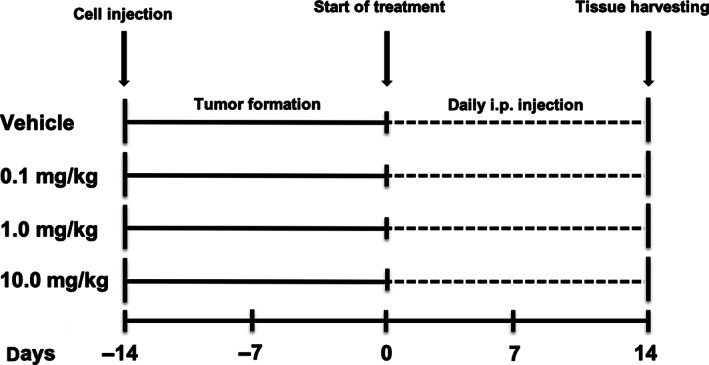
Animal study timetable. Tumor cell injection performed on left and right backsides in noncastrated mice. Tumor formation was generally observed after 14 days. Afterward, mice were divided into four groups: four animals were treated with daily intraperitoneal injection of vehicle, four with 0.1 mg/kg Dutasteride, four with 1.0 mg/kg Dutasteride and four with 10.0 mg/kg Dutasteride for 14 days

**FIGURE 2:**
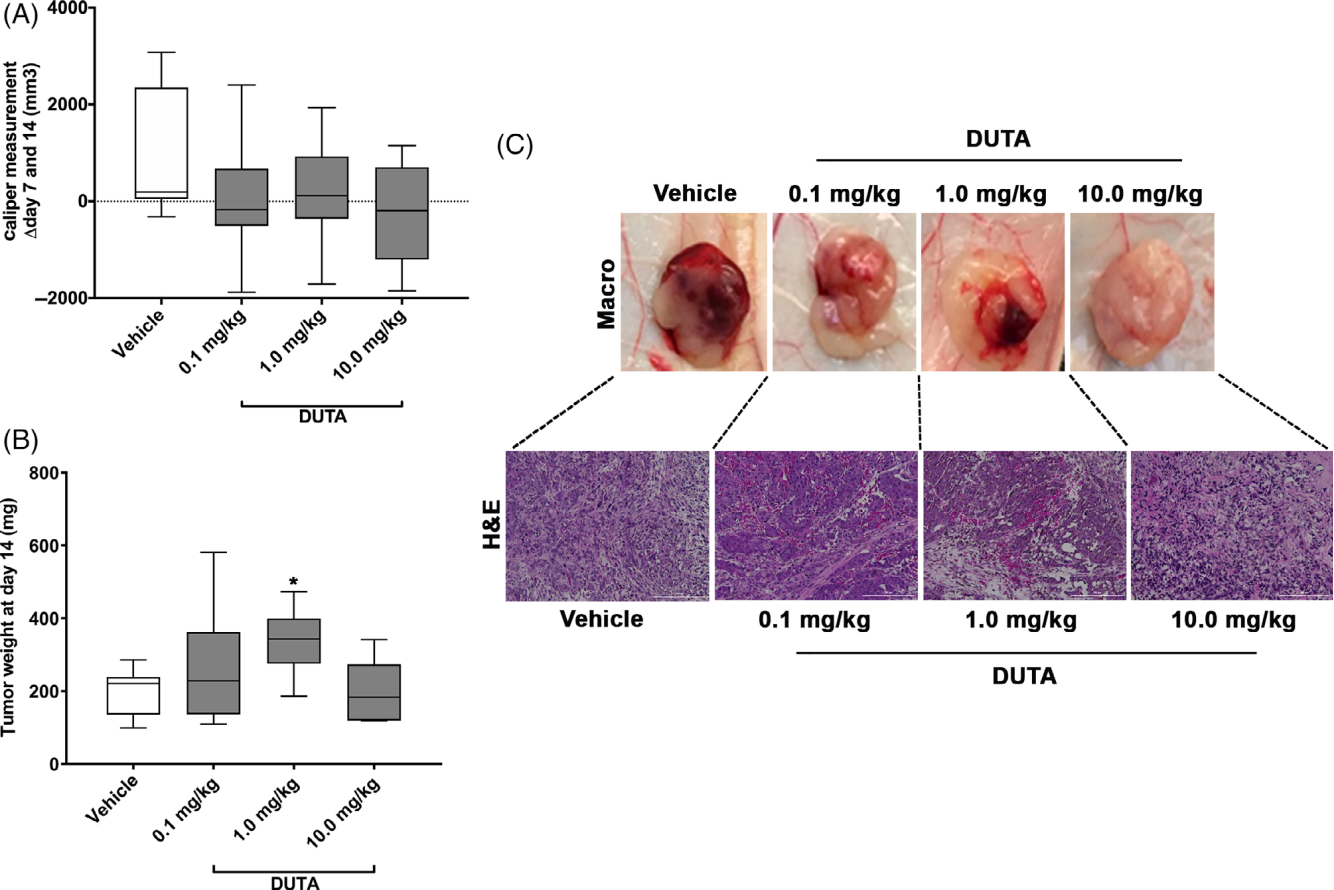
Tumor volume and weight. Tumor caliper measurement at day 7 and day 14 prior (A) and total tumor weight following tissue harvesting (B) in all four groups of mice treated with either daily intraperitoneal injections of vehicle, 0.1, 1.0, and 10.0 mg/kg Dutasteride for 14 days. Data are shown as minimum to maximum of six to eight measurements. Statistical analysis was performed using Kruskal‐Wallis test. *P < 0.05, **P < 0.01, ***P < 0.001, ****P < 0.0001. Representative macroscopic pictures and corresponding hematoxylin and eosin‐stained sections of formed tumor from all experimental conditions showing proper tumor formation (C)

A time‐dependent tumor growth was observed in all 16 mice within 14 days following injection of LNCaP cells before the first treatment (Figure 1). Caliper measurements were performed at day 7 and 14 following start of treatment with Dutasteride. Treatment with Dutasteride led to a nonsignificant reduction of mean tumor volumes in all treatment groups compared to vehicle control (Figure 2A). The caliper measurements revealed the following mean differences in tumor volume ± SEM between day 7 and day 14 (Δd7 ‐ d14): treatment with 0.1 mg/kg, 70 mm^3^ ± 436 mm^3^; treatment with 1 mg/kg, 154 mm^3^ ± 381 mm^3^; treatment with 10 mg/kg, −253 mm^3^ ± 438 mm^3^; vehicle control, 867 mm^3^ ± 474 mm^3^. All tissues formed by subcutaneously injected LNCaP cells were harvested after 14 days for analysis. Mean tumor weight at day 14 following treatment with Dutasteride was highest in the group treated with 1 mg/kg daily i.p. injections (335 mg ± 32 mg) and was significantly higher compared to vehicle control (201 mg ± 22 mg, *P* < 0.05). Groups treated with 0.1 mg/kg (264 mg ± 55 mg) or 10 mg/kg (199 mg ± 36 mg) showed no significant difference in mean tumor weight compared to vehicle control (Figure 2B). Proper tumor formation was confirmed by H&E stainings performed on representative samples from all experimental conditions (Figure 2C).

### 3.2 PSMA and AR expression following treatment with Dutasteride

**FIGURE 3:**
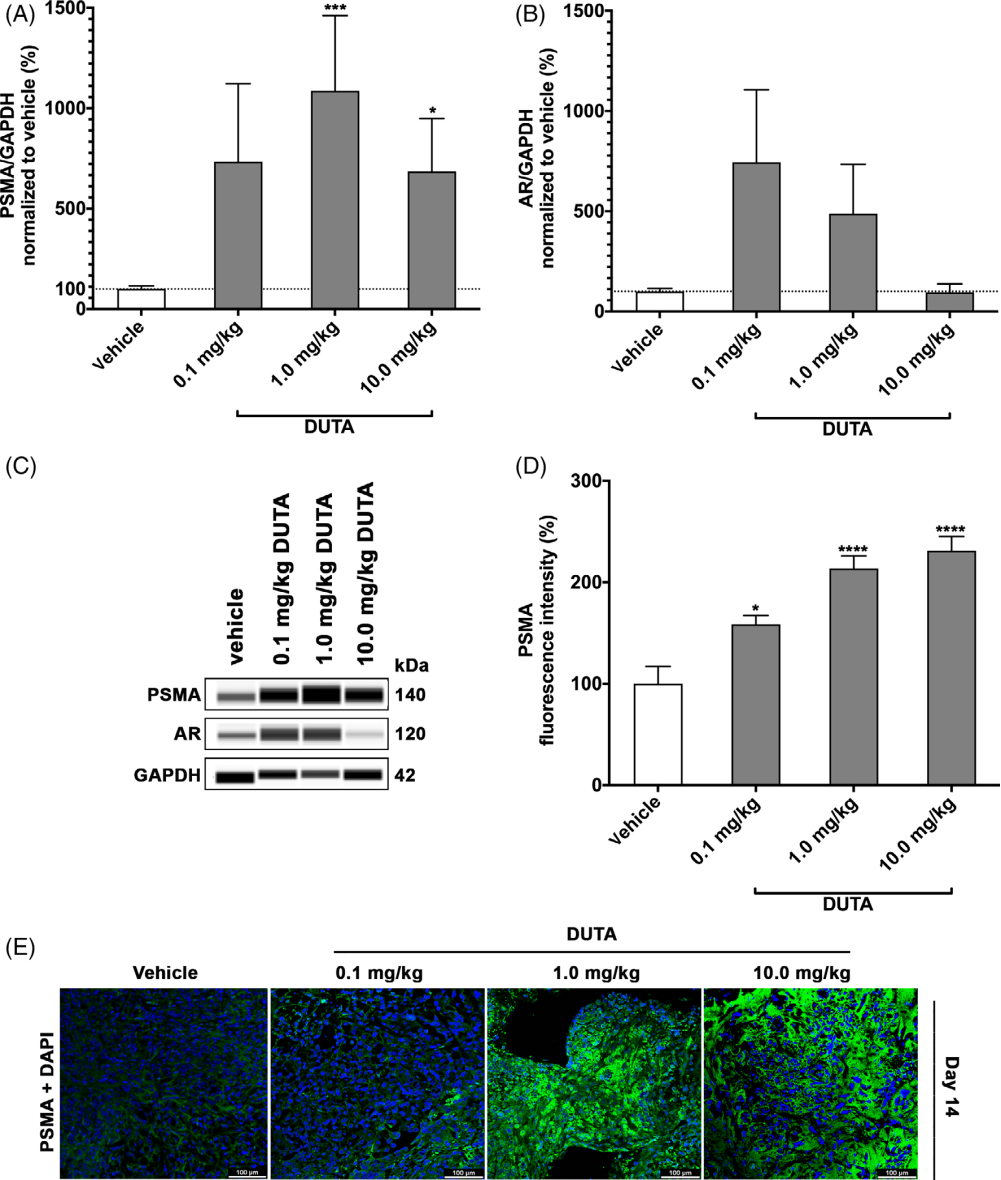
PSMA and AR expression. Total PSMA protein expression (A) and total AR protein expression (B) were measured on harvested tumor tissue from mice treated with daily intraperitoneal injections of vehicle, 0.1 mg/kg Dutasteride, 1.0 mg/kg Dutasteride, and 10.0 mg/kg Dutasteride for 14 days. PSMA and AR expression is presented as percentage compared to vehicle control. Data are shown as mean ± SE of the mean (SEM) of three to six independent experiments. Statistical analysis was performed using Kruskal‐Wallis test. *P < 0.05, **P < 0.01, ***P < 0.001, ****P < 0.0001. Protein simple immunoblotting (WES) from one representative experiment (C). PSMA fluorescence intensity was measured and quantified using image J software. The results are presented as percentage of PSMA expression compared to vehicle control. Data are shown as mean ± SE of the mean (SEM) of five to seven measurements. Statistical analysis was performed using ANOVA test. *P < 0.05, **P < 0.01, ***P < 0.001, ****P < 0.0001 (D). Visualization of PSMA expression using immunohistochemistry (E). Representative confocal images of PSMA surface staining. Tumor tissue sections were stained with primary anti‐PSMA antibody and detected using FITC (green) conjugated secondary antibody and DAPI (blue, 4′,6‐diamidino‐2‐phenylindole)

To analyze total PSMA and AR expression in tumor tissue following treatment with different concentrations of Dutasteride, we performed quantitative protein immunoblotting (Figure 3A‐C). A significantly upregulated mean PSMA protein expression was observed in the groups treated with 1 mg/kg (1087% ± 374%, *P* < 0.001) and 10 mg/kg (685% ± 263%, *P* < 0.05) Dutasteride, respectively, compared to mice treated with vehicle only. In the group treated with 0.1 mg/kg, no statistically significant PSMA alteration was noted (484% ± 239%) (Figure 3A). The increase of the mean AR expression was not significant in all Dutasteride treatment groups (0.1 mg/kg: 620% ± 321%; 1 mg/kg: 488% ± 246%; 10 mg/kg: 95% ± 42%) (Figure 3B).

In addition, immunohistological PSMA staining and quantification of PSMA fluorescence intensity were performed (Figure 3C,D,E). Compared to vehicle control, the mean fluorescence intensity increased significantly following all treatments with 0.1 mg/kg (159% ± 9%), 1 mg/kg (214% ± 12%), and 10 mg/kg (231% ± 14%) Dutasteride, respectively.

### 3.3 Caspase‐3, cleaved caspase‐3, and Ki‐67 expression following Dutasteride treatment

**FIGURE 4:**
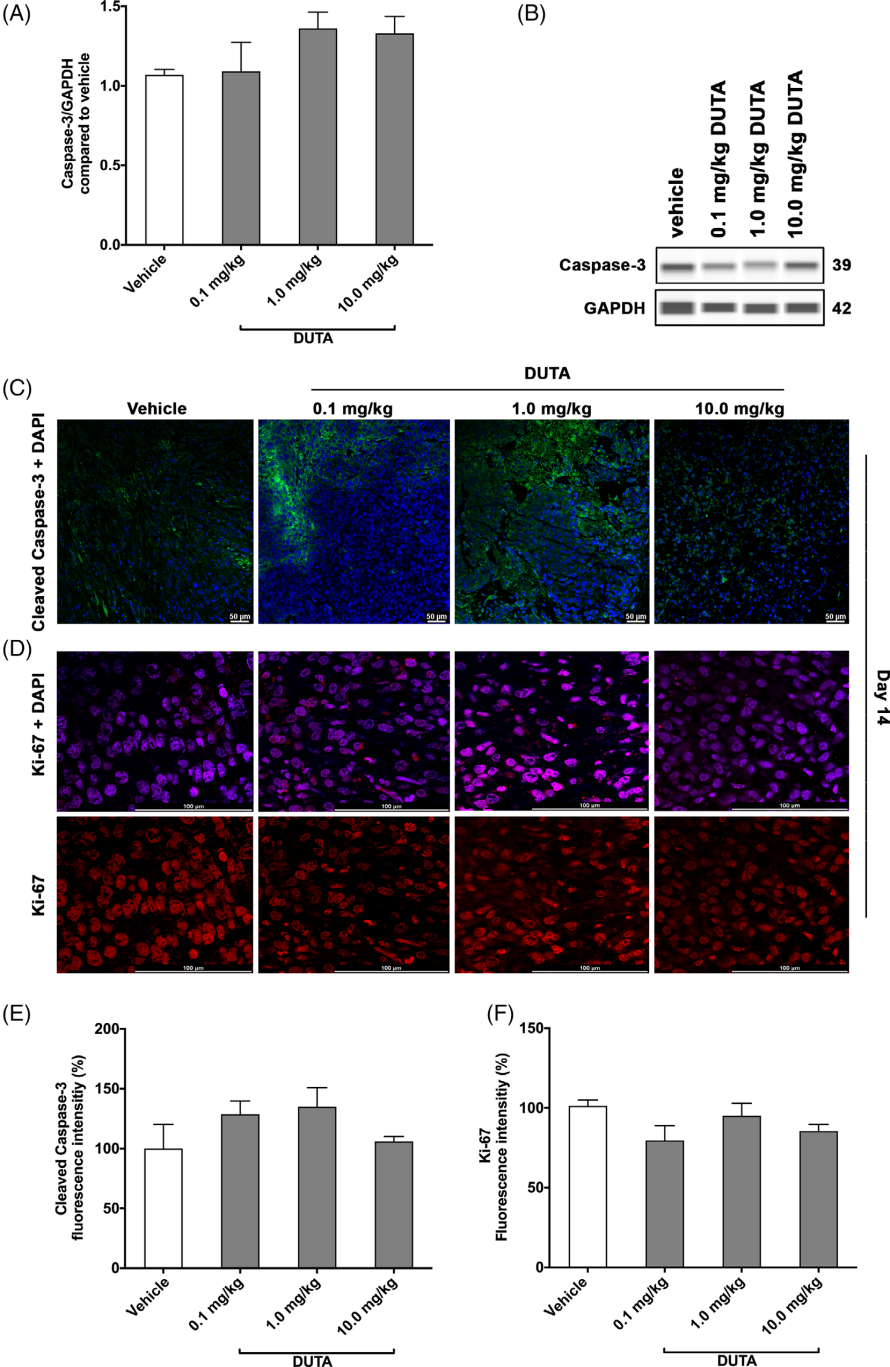
Protein expression pattern of caspase‐3, cleaved caspase‐3, and Ki‐67 in tumor tissues. Total caspase‐3 protein expression (A) measured on harvested tumor tissue from mice treated with daily intraperitoneal injections of vehicle, 0.1 mg/kg Dutasteride, 1.0 mg/kg Dutasteride, and 10.0 mg/kg Dutasteride for 14 days. Protein simple immunoblotting (WES) from one representative experiment (B). Representative immunofluorescent staining of tumor sections from all experimental conditions after 14 days of treatments, stained with cleaved caspase‐3 (FITC, green color) (C) and Ki‐67 (CY3, red color) (D). All samples were counter‐stained with DAPI (blue, 4′,6‐diamidino‐2‐phenylindole). Fluorescence intensity was measured and quantified using image J software. The results are presented as percentage of cleaved caspase‐3 (E) and Ki‐67 (F) expression compared to vehicle control. Data are shown as mean ± SE of the mean (SEM) of 15 to 20 measurements. Statistical analysis was performed using ANOVA test. *P < 0.05, **P < 0.01, ***P < 0.001, ****P < 0.0001

To analyze possible changes of apoptosis following Dutasteride treatment, a caspase‐3 immunoblotting was performed (Figure 4A,B). Treatment with different concentrations of Dutasteride did not significantly alter apoptosis compared to vehicle control. In mice treated with 0.1 mg/kg Dutasteride, the mean normalized apoptosis was 1.1 ± 0.2 compared to 1.1 ± 0.1 in mice treated with vehicle control. In the groups treated with 1 and 10 mg/kg Dutasteride, a mean apoptosis of 1.4 ± 0.1 and 1.3 ± 0.1 was observed, respectively. Immunohistological staining and quantification of cleaved caspase‐3 and Ki‐67 fluorescence intensity revealed nonsignificant changes following treatment with 0.1, 1, and 10 mg/kg Dutasteride (cleaved Caspase‐3:129% ± 11%, 135% ± 16%, 106% ± 4%; Ki‐67:80% ± 9%, 95% ± 8%, 85% ± 4%) compared to vehicle control (Figure 4C‐F).

## 4 DISCUSSION

In this in vivo study, we demonstrate for the first time that daily intraperitoneal injections of 1 and 10 mg/kg of Dutasteride in an LNCaP‐bearing mouse xenograft model significantly increase total PSMA protein expression. PSMA fluorescence intensity was significantly increased using even lower concentrations of 0.1 mg/kg Dutasteride daily. In addition, we show that Dutasteride does not significantly alter AR, caspase‐3, cleaved caspase‐3, and Ki‐67 expression in vivo.

Applications targeting PSMA especially for imaging, but also for therapy of prostate cancer, is increasingly used. However, the exact biologic function and regulation of PSMA is not yet fully understood.^16^ It has been shown that glycosylation is necessary for the enzymatic activity of PSMA, and that after internalization, it follows intracellular pathways to the endosomal compartment.^17^, ^18^, ^19^ Furthermore, in vitro data demonstrated that PSMA cross‐linking activates AKT, mTOR, and MAPK pathway.^20^ A recent in vivo study supported these data by showing that PSMA activates AKT signaling through the messenger molecule glutamate.^21^

For the first time, Wright et al reported upregulation of PSMA expression observed in matched pre‐ and posttreatment tissue specimens from 20 patients following initiation of ADT.^6^ Later, Murga et al suggested a time‐dependent effect of AR inhibition on PSMA expression using Enzalutamide in LNCaP and C4‐2 cells.^7^ Similar results were observed in VCaP cells treated with Abiraterone.^9^ Luckerath et al analyzed the effect of ADT on PSMA expression and its potential additive effect in combination with radioligand therapy in a C4‐2 xenograft mouse model and observed an increased PSMA expression following treatment with ADT. However, increased PSMA expression did not lead to a synergistic treatment effect when combined with radioligand therapy.^8^

Apart from preclinical data, a feasibility study including five patients suggested a possible PSMA enhancing effect using ADT for a median of 9 days (range 6‐11 days).^22^ Rosar et al demonstrated an increase of PSMA expression of 45‐55% in 10 patients planned for radioligand therapy scanned 2‐3 weeks following treatment start with Enzalutamide.^11^ However, due to small patient cohorts, definitive conclusions cannot be drawn. Others reported inconclusive prospective data in 9 men scanned 3‐4 weeks following start of treatment with ADT.^12^ Further, retrospective data from patients scanned 3 months following start of treatment with ADT could not demonstrate any significant PSMA alterations, indirectly indicating that a possible PSMA flare up phenomenon might reach its maximum after 2‐4 weeks.^10^, ^23^

While the influence of molecules altering the AR axis on PSMA expression is increasingly established, we were able to demonstrate that also Dutasteride significantly increases PSMA expression in vitro.^13^ Dutasteride is a 5‐alpha‐reductase inhibitor with a well‐known risk profile used for the treatment of lower urinary tract symptoms due to benign prostatic enlargement.^15^ Dutasteride inhibits 5‐alpha‐reductase‐isoenzymes type 1 and 2, regulating the synthesis of dihydrotestosterone from testosterone. Further research showed a concentration‐ and time‐dependent effect of Dutasteride on PSMA expression and uptake of ^177^Lu‐PSMA‐617 in vitro.^14^ In our current study, we could translate previously observed in vitro results into an LNCaP‐bearing mouse xenograft model, which was used due to its stable and well‐studied PSMA expression. We observed a significant 10‐ and 6‐fold increased mean PSMA protein expression in mice treated with 1 and 10 mg/kg Dutasteride, respectively. A lower PSMA protein expression in the 10 mg/kg compared to the 1 mg/kg group might reflect a saturation effect. In the group treated with 0.1 mg/kg Dutasteride, a nonsignificant fourfold increase was observed. Mean fluorescence intensity was significantly increased 1.6‐fold already at the low treatment concentration of 0.1 mg/kg Dutasteride and twofold using 1 and 10 mg/kg Dutasteride.

We tested a wide range of concentrations from 0.1 to 10 mg/kg applied by daily intraperitoneal injections over a period of 14 days. All concentrations have previously been used by other groups and reflect clinical applicable as well as higher concentrations. These concentrations of Dutasteride used in vivo are lower than those reported by the group of Litim et al, who studied the effect of Dutasteride on dopamine neurons in a mouse model of Parkinson's disease. Our test concentrations are comparable to those reported by Ateeq et al, who assessed the effect of Dutasteride in combination with Bicalutamide on tumor growth in a prostate cancer mouse model.^24^, ^25^ A direct comparison of these concentrations tested in vivo to concentrations generally used in patients is not possible since metabolism and administration route differ essentially.

In 2018, Kaittanis et al observed smaller tumors in a similar LNCaP bearing mice xenograft model following treatment with the highly selective PSMA inhibitor 2‐PMPA, supporting the hypothesis that PSMA might drive pro‐oncogenic activities.^21^ Others reported that Dutasteride in combination with the antiandrogen Bicalutamide significantly reduced the tumor burden in a VCaP‐bearing mouse xenograft model.^25^ In our study, we observed a nonsignificantly reduced tumor volume following treatment with high concentrations of Dutasteride. No significant changes in caspase‐3, cleaved caspase‐3, and Ki‐67 expression were observed.

In view of the low risk of severe side effects, Dutasteride can be considered an interesting candidate to potentially induce PSMA expression prior to PSMA targeting applications. Especially patients with early biochemical recurrence and planned salvage radiotherapy might benefit from enhanced detection and localization of recurrent prostate cancer foci. In addition, therapeutic effects might be increased in patients with advanced prostate cancer and planned radioligand therapy. However, heterogeneous tumor PSMA expressions potentially involving heterogenous regulation mechanisms, such as observed by Current et al, might represent a limitation for clinical applications.^26^

## 5 CONCLUSION

In conclusion, short‐term treatment with Dutasteride significantly increases total PSMA protein expression in an LNCaP‐bearing mouse xenograft model. Further investigations are needed to elucidate the impact of Dutasteride treatment on PSMA expression in prostate cancer patients.

## CONFLICT OF INTEREST

The authors declare no conflicts of interest.

## AUTHOR CONTRIBUTIONS

**Benedikt Kranzbühler:** Conceptualization; data curation; formal analysis; funding acquisition; investigation; methodology; project administration; writing‐original draft. **Rosa Sousa:** Investigation; writing‐review & editing. **Lukas Prause:** Investigation; writing‐review & editing. **Irene Burger:** Investigation; writing‐review & editing. **Niels Rupp:** Investigation; writing‐review & editing. **Tullio Sulser:** Investigation; writing‐review & editing. **Souzan Salemi:** Investigation; methodology; supervision; writing‐original draft. **Daniel Eberli:** Conceptualization; data curation; supervision; writing‐original draft.

## ETHICS STATEMENT

All animal experiments were performed in accordance with relevant institutional and national guidelines for the care and use of laboratory animals. Local animal ethical committee (Veterinäramt Zürich, Amendment to Licence No.244/2016) approved all experiments.

## Data Availability

The data that support the findings of this study are available from the corresponding author upon reasonable request.
